# Supplementary material for: Heat stress induces phage tolerance in Enterobacteriaceae
Source: eLife. 2025 Jul 7;14:RP105703. doi: 10.7554/eLife.105703 (PMC12234005; doi:10.7554/eLife.105703)
Supplement: Supplementary file 1. [file elife-105703-supp1.docx]

**Table S1. List of bacterial strains and phages used in this study.**

| Strain Name | Description | Reference |
| --- | --- | --- |
| ***K. pneumoniae*** |  |  |
| ATCC 43816 |  |  |
| *K381*  *K357* | Isolated from Hospital  Isolated from Hospital | This Study  This Study |
| *pspA** | Evolution BC2G11C1 | This Study |
| *rpoH** | Evolution BC3G11C2 | This Study |
| *Δcps* | ATCC 43816 *Δcps* | (Huang et al., 2022) |
| *ΔpspA* | ATCC 43816 *ΔpspA* | This Study |
| ***E. coli*** |  |  |
| *KLY* | KL16-YFP Cam | (Fridman et al., 2014) |
| *metG** | KLY *metG^T^* | (Levin-Reisman et al., 2017) |
| **Phages** |  |  |
| Kp11 | *K. pneumoniae* phage (GenBank: ON148528.1) | Provided by Xiang Lab.(Huang et al., 2024) |
| Kp7 | *K. pneumoniae* phage (GenBank: ON148527.1) |  |
| Kp9 | *K. pneumoniae* phage (GenBank: ON148529.1) |  |
| Kp10 | *K. pneumoniae* phage |  |
| T1/T4 | MG 1655 *E. coli* phage | Provided by Liang Lab. |
