# Supplementary material for: Heat stress induces phage tolerance in Enterobacteriaceae
Source: eLife. 2025 Jul 7;14:RP105703. doi: 10.7554/eLife.105703 (PMC12234005; doi:10.7554/eLife.105703)
Supplement: Supplementary file 2. [file elife-105703-supp2.docx]

**Table S2. List of plasmids and primers used in this Study.**

| Primer name | Description | Reference |
| --- | --- | --- |
| pCasKP-apr | Thermosensitive plasmid carrying Cas9 gene and lambda red system; Apr^R^ | (Wang et al., 2022)Provided By Ji Lab. |
| pSGKP-spe | Expressing sgRNA and carrying *sacB*; Spe^R^ |  |
| **Primers** |  |  |
| ZF-pspA1 | tagtGCAGTTTGCCGAACTGAAAGCGG | This Study  For pspA deletion |
| ZF-pspA2 | aaacCCGCTTTCAGTTCGGCAAACTGC |  |
| ZF-pspA3 | TGTCGCCAGCTCATCGAGAAACAGCGTC |  |
| ZF-pspA4 | TTTCTCGATGAGCTGGCGACA |  |
| ZF-pspA5 | TGAAACCAGAACTATGAGGATTGAAATTGTATGATTGCGCGGCGCGGCGTTGCG |  |
| ZF-pspA6 | ATGAGCTCCACCGCGGTGGCGGCCGCTCTAGCCGTACAGCTCGCTGTCCGGTAT |  |
| ZFqP1-F | GACGATCCCTAGCTGGTCTG | This Study  For qPCR |
| ZFqP1-R | GTGCAATATTCCCCACTGCT |  |
| ZFqP2-F | GACATCCCGTTCGACTACCT |  |
| ZFqP2-R | GCAGCTGTGAGACGTTAAGG |  |
